# Heating capabilities of small fluid warming systems

**DOI:** 10.1186/s12871-018-0565-x

**Published:** 2018-07-28

**Authors:** Norbert Zoremba, Christian Bruells, Rolf Rossaint, Thomas Breuer

**Affiliations:** 1 Department of Anaesthesiology, Sankt Elisabeth Hospital Gütersloh, Stadtring Kattenstroth 130, D-33332 Gütersloh, Germany; 2 Department of Anaesthesiology, Medical Faculty, RWTH Aachen University, Pauwelsstrasse 30, D-52074 Aachen, Germany; 3 Department of Intensive and Intermediate Care, Medical Faculty, RWTH Aachen University, Pauwelsstrasse 30, D-52074 Aachen, Germany

**Keywords:** Hypothermia, Infusion, Body temperature, Anesthesia, Fluid warmer

## Abstract

**Background:**

Perioperative temperature management is fundamental to ensure normothermia in patients. Fluid warmers, which have become smaller in size over the past few years, can help to maintain a stable body temperature. Potentially, the reduction of the size may influence the heating performance.

**Methods:**

Therefore, we tested the effectiveness of enFlow®, Fluido compact® and Thermosens® fluid warmers by measuring the inlet and outlet temperature for room-tempered and ice-cooled saline at flow rates of 25, 50, 75 and 100 ml/min.

**Results:**

At all examined flow rates, the tested heating devices warmed up room-tempered saline effectively. The enFlow® provided the significantly (*p* < 0.05) highest outlet temperature throughout all tested flow rates in comparison to the other devices. When ice-cooled saline was used, the enFlow® maintained a stable outlet temperature > 38 °C at all tested flow rates. The Fluido compact® ensured this only at flow rates of 25 and 50 ml/min, while the Thermosens® provided these conditions at flow rates of 25, 50 and 75 ml/min.

**Conclusions:**

The heating capability for room-tempered saline was effective in all tested devices, but with ice-cooled saline enFlow® is superior at high flow rates. At low flow rates the heating capabilities of enFlow®, Fluido compact® and Thermosens® are comparable.

## Background

Maintaining a constant body temperature during anaesthesia is very important to prevent major complications and prolonged hospitalisation [1–3]. Therefore, a sufficient temperature management in patients must be a major target during operative procedures. A drop of body core temperature below 36 °C matches the criterion of hypothermia [4]. To prevent such a decline, several devices exist in the operating room to maintain normothermia. Common systems use conductive and convective temperature regulation or pre-warmed infusions [5, 6]. The warming of infusion fluids has a clinical impact, because the infusion of cold saline or packed red cells causes a significant decrease in core temperature. It is possible to estimate energy expenditures related to the supply of cold fluids. To increase the body temperature of 1 kg of water by 1 °C, 1 kcal of energy is needed. Thus, the patient has to provide 16 kcal of energy to increase the temperature of 1 l crystalloids from room temperature (21 °C) to body temperature of 37 °C [7]. The common way of delivering warmed fluids is either a double-wall infusion line or heating the fluid in a warming device above body temperature because it will cool down until reaching the patients’ venous system [8, 9]. Small heating devices use boxed heating plates, which can be placed close to the patient reducing the length of the infusion line between device and patient. This length reduction may have a significant influence on the temperature of the infused solution. By minimizing the size, small heating devices have a reduced warming area causing a limitation of heating capacity based on flow and temperature of the infused fluids. The outcome of this may be a reduced warming ability at higher flow rates and with precooled solutions.

The study intends to compare the heating capabilities of the small fluid warming systems enFlow®, Fluido compact® and Thermosens® in various experimental settings. These three devices were tested at different flow rates with both precooled and room-tempered solutions.

## Methods

Heating properties of the enFlow® (Carefusion, Vernon Hills, IL, USA), the Fluido Compact® (The 37° Company, Amersfoort, Netherlands), and Thermosens® (Barkey, Leopoldshöhe, Germany) were compared in defined experimental settings according to the manufacturers` instructions. A series of measurements was performed using isotonic sodium chloride (Braun, Melsungen, Germany) at room temperature, followed by cooled saline. Precooled solutions were stored in a regular laboratory refrigerator for 48 h and put in an ice bath during the measurements. The infusion was connected to a roller pump (CAPS, Stoeckert Instrumente GmbH, Munich, Germany) to maintain a constant infusion flow, following a connection to the fluid warming system. The length of the infusion line between the roller pump and the heating device was 10 cm. The heating devices were filled with saline and connected to the inlet and outlet tubing system. According to the different devices the outlet tubing differs in length and diameter. To ensure similar experimental conditions the outlet tubing was shortened if necessary and connected to a defined outlet tubing. In total, the outlet tubing had a length of 100 cm. Temperature probes (ML309 Thermistor Pod**,** AD instruments, Oxford, UK) were placed directly in front of and behind the heating chamber. Additionally, the temperature was measured at a distance of 50 cm and 100 cm behind the fluid warming system. Each probe was inserted via a 3-way stopcock and a non-return valve and connected to a Powerlab device (ADinstruments, Oxford, UK). The temperature was recorded with a sample rate of 1000 Hz (LabChart software Version 7.3.4, ADinstruments, Oxford, UK). Before the start of measurements at defined infusion flows (25, 50, 75 and 100 ml/min), the exactness of the roller pump flow rate was tested using a measurement cylinder. Thereafter, five recordings were preformed with an interruption of 30 s between each measurement in order to maintain comparable heating conditions.

### Statistics

The results of the experiments are expressed as the mean ± standard deviation (SD). Statistical analysis of the differences within and between groups was calculated using two-way ANOVA. If any significance was detected (*p* < 0.05), a Bonferroni post-hoc test was applied. The differences between the inlet and outlet temperature (ΔT) were calculated using one-way ANOVA, followed also by a Bonferroni post-hoc test, if any significance was detected (Prism 6, GraphPad Software, La Jolla, CA, USA).

## Results

### Room-tempered saline warming capability

**Fig. 1:**
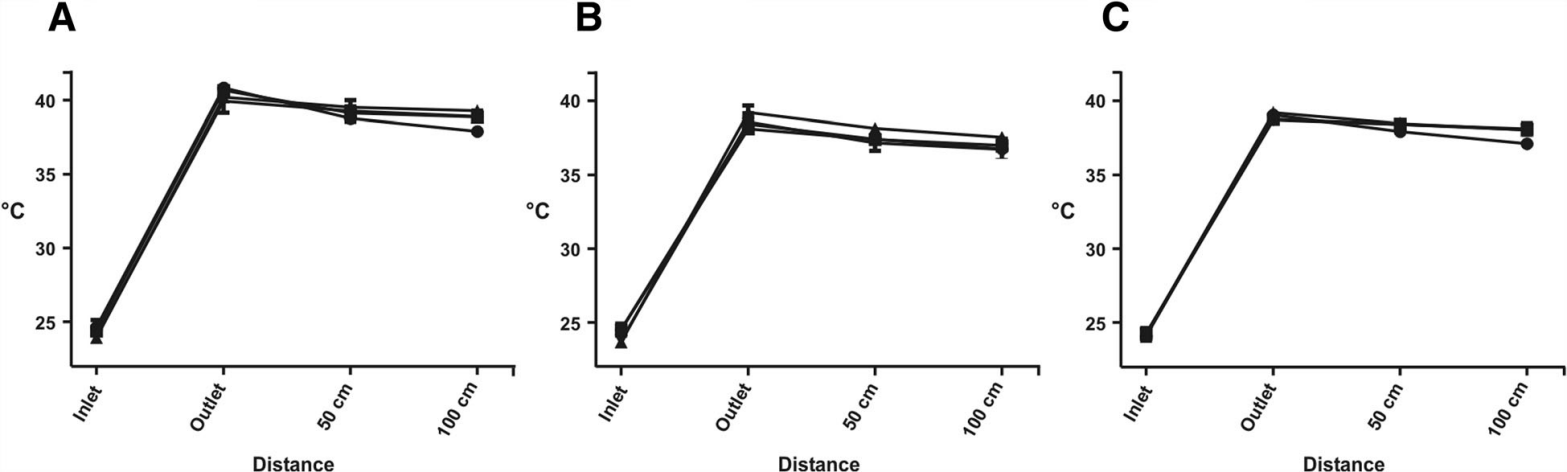
Mean temperature measured in front of (inlet), directly (outlet), 50 and 100 cm behind the heating device for room-tempered saline. The tested flow rates for the en-Flow (**a**), Fluido (**b**) and Thermosens (**c**) were 25 ml/min (●), 50 ml/min (■), 75 ml/min (▲) and 100 ml/min (▼). Error bars represent SD

In the row of experiments with room-tempered saline, the fluid temperature when entering the device was 24.2 ± 0.4 °C (enFlow®), 24.1 ± 0.5 °C (Fluido Compact®) and 24.2 ± 0.3 °C (Thermosens®). There was no statistical difference in the inlet temperature between the tested devices throughout the different flow rates (Fig. 1a-c). At a flow rate of 25 ml/min and 50 ml/min, the outlet temperature of enFlow® (40.8 ± 0.3 °C, 40.7 ± 0.4 °C) was significantly higher (*p* < 0.01) than the ones of Fluido Compact® (38.4 ± 0.3 °C, 38.1 ± 0.3 °C) and Thermosens® (39.1 ± 0.2 °C, 38.8 ± 0.2 °C). There was also a significant difference between Fluido Compact® and Thermosens® (*p* < 0.05) at these flow rates (Fig. 1). At a flow rate of 75 ml/min and 100 ml/min, the outlet temperature of the enFlow® (40.2 ± 0.3 °C, 39.9 ± 0.8 °C) was significantly higher (*p* < 0.01 at 75 ml/min and *p* < 0.05 at 100 ml/min) than the ones of Fluido Compact® (39.2 ± 0.5 °C, 38.6 ± 0.6 °C) and Thermosens® (39.2 ± 0.2 °C, 38.7 ± 0.4 °C). No statistical difference was found at flow rates of 75 ml/min and 100 ml/min between Fluido Compact® and Thermosens®. For the comparison of the warming capacities, the difference between the inlet and outlet temperatures t_outlet_ – t_inlet_ (ΔT) was calculated. We found a statistically significant higher ΔT in enFlow® than in Fluido Compact® (*p* < 0.01 at 25 and 50 ml/min, *p* < 0.05 at 100 ml/min). At all flow rates we also found a higher ΔT in enFlow® than in Thermosens® (p < 0.01 at 25, 50 and 75 ml/min, *p* < 0.05 at 100 ml/min). However, at flow rates of 25 ml/min and 50 ml/min significantly higher ΔT (*p* < 0.01 and *p* < 0.05, respectively) was measured in Thermosens® than in Fluido Compact®.

### Precooled saline warming capability

**Fig. 2:**
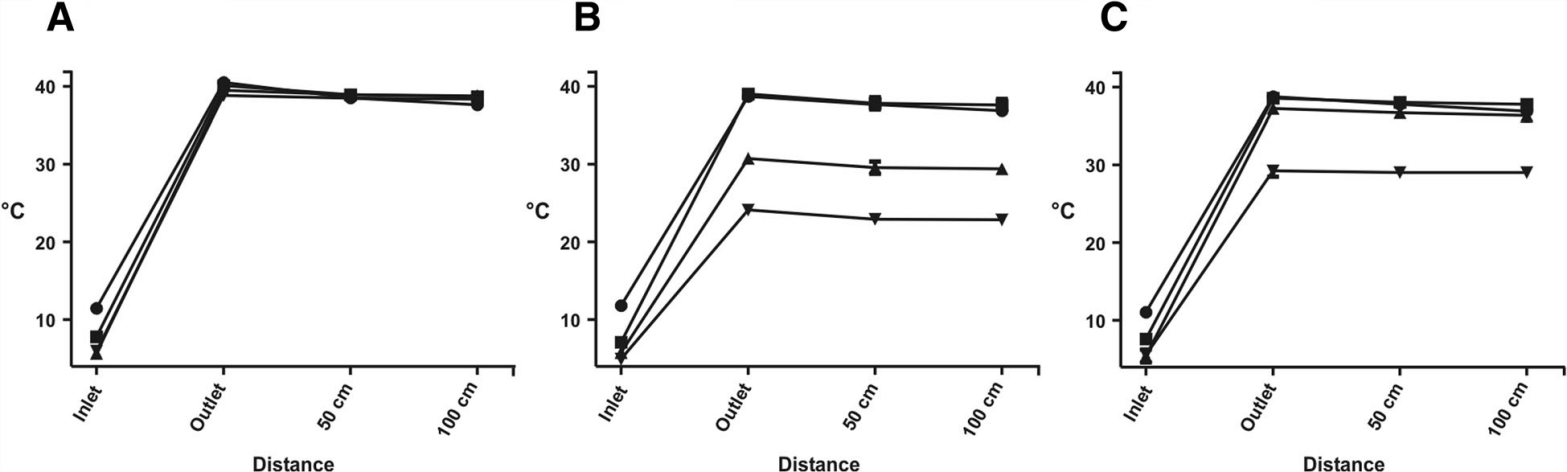
Mean temperature measured in front of (inlet), directly (outlet), 50 and 100 cm behind the heating device for ice-cooled saline. The tested flow rates for the en-Flow (**a**), Fluido (**b**) and Thermosens (**c**) were 25 ml/min (●), 50 ml/min (■), 75 ml/min (▲) and 100 ml/min (▼). Error bars represent SD

In the measurements with precooled saline at flow rates of 25, 50, 75 and 100 ml/min, the fluid temperature entered the device was 11.5 ± 0.2 °C, 7.8 ± 0.3 °C, 5.7 ± 0.6 °C and 5.9 ± 0.3 °C (enFlow®), 11.8 ± 0.2 °C, 7.1 ± 0.4 °C, 5.8 ± 0.5 °C and 5.0 ± 0.8 °C (Fluido Compact®) and 11.1 ± 0.5 °C, 7.6 ± 0.6 °C, 5.4 ± 0.8 °C and 5.5 ± 0.3 °C (Thermosens®), respectively. Consequently, there was no statistical difference in the inlet temperature between the devices at comparable flow rates (Fig. 2a-c). Considering the flow rates of 25, 50, 75 and 100 ml/min the outlet temperature of enFlow® (40.5 ± 0.3 °C, 40.1 ± 0.7 °C, 39.5 ± 0.2 °C, 38.8 ± 0.4 °C) was significantly higher (*p* < 0.01) than of Fluido Compact® (38.7 ± 0.5 °C, 39.0 ± 0.3 °C, 30.7 ± 0.3 °C, 24.1 ± 0.2 °C) and Thermosens® (38.8 ± 0.3 °C, 38.6 ± 0.5 °C, 37.2 ± 0.5 °C, 29.4 ± 0.7 °C). The Thermosens® outlet temperature was significantly higher (*p* < 0.01) than Fluido Compact®´s only at flow rates of 75 and 100 ml/min. At a flow rate of 25, 75 and 100 ml/min, the ΔT of enFlow® was significantly higher (*p* < 0.01) than of Fluido Compact® and Thermosens®. At these flow rates, we also found significantly higher (*p* < 0.01) ΔT in Thermosens® compared to Fluido Compact®. At a flow rate of 50 ml/min, however, we did not detect any significant differences in ΔT between the tested devices. In each measurement the saline temperature declined significantly with increasing distance to the heating device.

## Discussion

We tested the warming capacity of small heating devices at flow rates of 25, 50, 75 and 100 ml/min. At all examined flow rates, all tested devices warmed up room-tempered saline effectively. Nevertheless, enFlow® provided the highest outlet temperature throughout all different flow rates. The heating performance differs among the tested devices when cooled fluid such as packed red blood cell infusion is used. While enFlow® maintained a stable outlet temperature > 38 °C at all tested flow rates, Fluido compact® ensured this only at flow rates of 25 and 50 ml/min. The Thermosens® showed a significantly lower outlet temperature only at flow rates of 100 ml/min. Therefore, the best heating performance with both room-tempered and cooled saline was, at all flow rates, achieved by the enFlow®. Although Fluido compact® and Thermosens® provide higher infusion temperatures compared to no warming, the maintenance of normothermia for prolonged periods may be limited when high infusion rates with cooled infusion fluids are necessary. Furthermore, the heating performance of these used devices has a limit of efficacy. It was shown that the efficacy of dry heat and water bath warmers has a limit of 33 °C [9]. Administering intravenous fluids at an infusion rate of 4 ml/kg/h is advantageous in reducing postoperative morbidity in abdominal surgery [10, 11]. At this infusion rate, Fluido compact® and Thermosens® administer adequatly warmed fluid, which is appropriate in a perioperative setting without major blood loss. If higher flow rates are needed, the enFlow® is capable to warm both room-tempered and ice-cooled saline effectively and is therefore a more flexible device during surgical procedures.

This study has some methodical limitations, which should be addressed. First of all, we tested the heating capabilities with saline. The infusion of packed red blood cells, frozen plasma or colloids may affect the warming conditions. In addition, we tested only two inlet temperatures. Room-tempered saline reflects a normal infusion temperature for crystalloids used in the operation setting, while ice-cooled saline mimics the clinical situation when packed red blood cells are infused. We did not test other inlet temperatures to detect the threshold inlet temperature for the restriction of the heating capabilities. Compared to the temperature exist in an operation room the room-temperature in our laboratory setting was slightly higher. This higher inlet temperature may enhance the heating performance of the heating devices. Likewise, we tested the heating devices at four fixed flow rates. In clinical practice, flow rates vary over the operation time. Thus, transferring our results directly to a clinical setting could be complicated. In the row of experiments with room-tempered solutions we measured stable temperatures before the heating devices. In the row of experiments with precooled solutions the inlet temperature at low flow rates was higher than at high flow rates. Reason for this is a beginning equalisation to room temperature over the 10 cm infusion line between roller pump and tested fluid warming system. Nevertheless, the results are comparable, because these conditions are equal for all tested heating devices.

## Conclusion

All tested devices facilitate an adequate warming at low flow rates. At higher flow rates and particularly with cooled infusion fluids only the enFlow® can maintain stable heating modalities. Therefore, enFlow® is superior when rapid infusion of cooled infusion fluids is necessary. If it is not, as for example in routine operations, the heating capabilities of enFlow®, Fluido compact® and Thermosens® are equally suitable.

## Abbreviations

cm: centimeter
m: meter
ml/min: mililiter per minute
SD: standard deviation
ΔT: Difference between inlet and outlet temperature
